# Assessing the potential of *DZIP1L* gene in autosomal recessive polycystic kidney disease gene therapy

**DOI:** 10.1002/pdi3.44

**Published:** 2023-11-01

**Authors:** Fahreddin Palaz

**Affiliations:** ^1^ Faculty of Medicine Hacettepe University Ankara Turkey; ^2^ Department of Medicine Massachusetts General Hospital, Harvard Medical School Boston MA USA

**Keywords:** AAV, ARPKD, *DZIP1L*, gene therapy, *PKHD1*

I read with interest the recent review by Lu et al. (Gene therapy for pediatric genetic kidney diseases. *Pediatr Discov*. 2023; 1(1):e16) and congratulate the authors for their comprehensive review.^1^ The authors stated that *PKHD1* is currently the only known disease‐causing gene for autosomal recessive polycystic kidney disease (ARPKD). However, while *PKHD1* is the primary gene implicated in ARPKD, it has been shown that *DZIP1L* is the second gene involved in the disease pathogenesis and is associated with a moderate form of ARPKD.^2^, ^3^, ^4^ The role of *DZIP1L* in ARPKD is also widely discussed in the review referenced by the authors to support their claim.^5^ Moreover, while it is a rarer cause of the disease, the *DZIP1L* gene has several advantages over *PKHD1* in the development of gene therapy for ARPKD.

In 2017, Lu et al. sequenced 743 patients presenting with suspected ARPKD or sporadic PKD. This investigation unveiled *DZIP1L* mutations as the causative factor underlying an ARPKD‐like phenotype in 7 patients from 4 consanguineous families.^2^ The study also demonstrated that the DZIP1L protein localized to centrioles and the basal bodies of primary cilia, and ciliary trafficking of polycystin‐1 and polycystin‐2 was impaired in *DZIP1L*‐mutant cells.^2^, ^3^ Concordantly, Hertz et al. recently reported 4 ARPKD patients with *DZIP1L* mutations from 3 consanguineous families.^4^

Given the extensive length of *PKHD1*, with its product named fibrocystin consisting of 4074 amino acids, it is not possible to package the cDNA of the gene into a single, or even dual, adeno‐associated virus (AAV) vector, which is the most commonly used vector for *in vivo* gene delivery for inherited diseases.^6^ Moreover, *PKHD1*‐associated ARPKD is often accompanied by congenital hepatic fibrosis, necessitating that any gene therapy developed for the disease would also target the biliary epithelium.^3^ Therefore, exceeding the packaging capacity of AAV vectors and the challenges of efficiently targeting both the liver and kidneys may hinder the progress of genomic medicines in treating *PKHD1*‐associated ARPKD.

Although being a rarer cause of the disease, the smaller size of the *DZIP1L* gene and its protein consisting of 767 amino acids offer a remarkable advantage for gene therapy applications by overcoming the AAV packaging limitations faced by *PKHD1*. Furthermore, liver involvement in the form of hepatosplenomegaly was reported for only one of the 11 patients with *DZIP1L* mutations, and the patients with truncating mutations showed no apparent hepatic complications.^2^, ^4^ While it is required to characterize more patients with diverse *DZIP1L* mutation profiles to establish phenotypic characteristics of *DZIP1L*‐associated ARPKD, the available data suggest that liver involvement in *DZIP1L*‐associated ARPKD might be less frequent and milder than *PKHD1*‐associated ARPKD. Therefore, it might be sufficient for patients with *DZIP1L* mutations to target the kidneys using local delivery routes, such as retrograde ureteral or renal vein injection, which may enable more efficient and specific gene delivery to the kidneys.^7^, ^8^

The potential for gene therapy in *DZIP1L*‐associated ARPKD will become more evident as new cohorts of patients with *DZIP1L* mutations are reported and the phenotypic features of the disease are characterized in more detail. Further research is warranted to fully assess the therapeutic potential of *DZIP1L*‐based gene therapy strategies and advance the development of genomic medicines for ARPKD.

## CONFLICT OF INTEREST STATEMENT

The author declares no competing interests.

## Data Availability

Data sharing not applicable to this article as no datasets were generated or analyzed during the current study.
